# Response of Rowan Berry (*Sorbus redliana*) Shoot Culture to Slow Growth Storage Conditions

**DOI:** 10.3390/plants12061287

**Published:** 2023-03-12

**Authors:** Nóra Mendler-Drienyovszki, Katalin Magyar-Tábori

**Affiliations:** Research Institute of Nyíregyháza, Institutes for Agricultural Research and Educational Farm (IAREF), University of Debrecen, P.O. Box 12, 4400 Nyiregyhaza, Hungary

**Keywords:** *Sorbus redliana* ‘Burokvölgy’, in vitro conservation, midterm preservation, tissue culture, cold storage, endangered species

## Abstract

Slow growth storage can preserve the genetic resources of endangered species such as those of genus *Sorbus*. Our aim was to study the storability of rowan berry in vitro cultures, their morpho-physiological changes, and regeneration ability after different storage conditions (4 ± 0.5 °C, dark; and 22 ± 2 °C, 16/8 h light/dark). The cold storage lasted for 52 weeks, and observations were made every four weeks. Cultures showed 100% survival under cold storage, and those taken from the storage showed 100% regeneration capacity after the passages. A dormancy period lasting about 20 weeks was observed, followed by intensive shoot growth until the 48th week, which led to the exhaustion of the cultures. The changes could be traced to the reduction of the chlorophyll content and the F_v_/F_m_ value, as well as in the discoloration of the lower leaves and the appearance of necrotic tissues. Long, etiolated shoots (89.3 mm) were obtained at the end of cold storage. Shoot cultures stored in a growth chamber as control (22 ± 2 °C, 16/8 h light/dark) senesced and died after 16 weeks. Explants from stored shoots were subcultured for four weeks. The number and length of newly developed shoots were significantly higher on explants from cold storage compared to those from control cultures if the storage was longer than one week.

## 1. Introduction

*Sorbus redliana* (common name: rowan berry) belongs to genus *Sorbus* (*Rosaceae*, *Maloideae*) [1]. *Sorbus* species are found throughout the northern temperate zone, where they are native to the natural forests. The genus is extremely rich in species and forms, and includes several interspecific hybrids [1,2]. *Sorbus* species and cultivars are important ornamental trees and medicinal plants [3]. They are deciduous trees or shrubs with lobed [4] or pinnately compound leaves [5]. They have pleasing umbel inflorescence and typically bloom in May. Their small apples are colored from white to yellow through red to brown. *Sorbus* trees maintain a long-lasting decorative appearance with their beautifully colored foliage and fruits which last into autumn [5].

Although a very small proportion of berry yield is currently utilized as raw materials in the products intended for human consumption, the bioactive compounds detected in *Sorbus* plants may make it more useful in the future for medicines, cosmetics, and functional foods [3]. *Sorbus* species can also be valuable raw materials for the wood industry [6].

*Sorbus redliana* is a rowan berry tree with a spherical crown and low plant height, which has branching from the base and grows into a large bush or a small tree of up to 5 m. The leaves are simple, ovate, wedge-shouldered, and dentate with a bright coloration and a downy lower surface. It is a spontaneous hybrid of *S. aria* and *S. torminalis*. Its white flowers bloom in May, with the ripe fruits being cinnabar-red and slightly warty. The autumn foliage color is yellow to brown. *S. redliana* ‘Burokvölgy’ is a Hungarian cultivar, native to one valley of the Bakony Mountains. It has tolerance to drought and is a decorative ornamental tree of gardens and streets [7].

Many *Sorbus* species have only a small number of individuals and are considered either critically endangered or vulnerable due to the harmful effects of climate change and habitat loss [8,9]. The special reproduction method of the genus *Sorbus*, including the easy creation of hybrids and polyploids (especially triploids) [10] often leads to the formation of small, fragmented populations that are particularly sensitive to adverse environmental effects, e.g., grazing, harmful forest management, etc. [9]. The purpose of gene conservation is to preserve genetic diversity and adaptability, which is a prerequisite for the long-term survival of species and populations [11,12]. The search for and preservation of valuable sources of disease resistance, such as fire blight-resistant genotypes in members of the *Rosaceae* family [13], is a vital role of current conservation efforts. One of the most common means of preserving valuable genetic material for seed-propagated crops is storage in the form of seeds at temperatures between −15 and −20 °C or between 0 and 4 °C [14].

Those species that are propagated vegetatively because they do not produce seeds or those with offspring whose genetic identity cannot be ensured with seeds may be best suited to storage with biotechnological methods, including tissue cultures used for micropropagation, slow growth methods, or cryopreservation [14,15,16]. These methods are particularly suitable for the conservation of genetic resources of the forest trees and horticultural plants [17], and the pathogen-free in vitro cultures can also be used effectively for the distribution of germplasm [18].

Slow growth storage (SGS) is a cost- and space-efficient method, suitable for midterm storage for months to up to several years [19]. The growth of in vitro cultures can be slowed down by changing the culture medium (e.g., less nutrients, sugar, modified growth regulator content, addition of osmotically active agents) and/or modified environmental factors (temperature and/or illumination reduction, even complete darkness) [15], or a combination of these variables [19]. This storage method most frequently takes the form of in vitro organ cultures—or even more recently alginate-encapsulated shoot explants [20] being kept at a low temperature (about 4 °C for temperate plant species and about 10–15 °C for tropical crops) [14]. With SGS, the number of subcultures is significantly reduced, and depending on the species, subcultures can even be extended for several years [15].

Guidelines describing the conservation of *Sorbus* species such as *S. domestica* and *S. cuneifolia* suggest that at least 50 individuals should be protected in situ, as these are among the most endangered forest tree species in the world [21,22,23]. Ex situ methods are of great importance, and these include conservation in the form of seeds (*S. domestica*) [24], the cryopreservation of shoot tips [25], in vitro tissue culture [26,27], and culture in cold storage conditions [28]. However, the morphological and physiological response of each plant species must be investigated to develop optimal in vitro culture and storage conditions [29].

The most suitable medium for *S. domestica* is the Schenck and Hildebrandt [30] medium supplemented with 22.2 µM of 6-benzylaminopurine (BA) [26]; the most suitable for *S. aucuparia* is the MS [31] medium with 0.9–1.8 µM of BA and 0.5 µM of indole-3-butyric acid (IBA) [32]; and the most suitable for *S. rotundifolia* is the MS medium containing 1.6 µM of IBA, 1.4 µM of 6-benzyladenine riboside (BAR), and 0.6 µM of gibberellic acid (GA_3_) [33]. An application of ½ MS medium supplemented with 3.3 µM BA, 0.5 µM IBA, and 0.3 µM GA_3_ has also been used effectively for *S. rotundifolia* [34]. Application of meta-topolin (2.1 µM) with IBA (0.5 µM) in MS medium was shown to be useful for micropropagation of *S. abscondita* and *S. omissa*, whereas *S. gemella* can be propagated on MS medium containing only meta-topolin (2.1 µM) as plant growth regulator [35]. However, a three- or four-week subculture period must be considered for micropropagated plants [36,37]; therefore, in order to preserve genetic resources more efficiently, it is advisable to use techniques that can significantly reduce the number of subcultures of in vitro cultures [37]. We studied the storage ability of in vitro shoot cultures of rowan berry (*Sorbus redliana* ‘Burokvölgy’) by monitoring their morpho-physiological changes during cold storage under 4 ± 0.5 °C and dark (4 °CD) and their viability after different lengths of cold storage compared to those left in a growth chamber under 22 ± 2 °C, 16/8 h light/dark conditions (22 °CL).

## 2. Results

### 2.1. Morphological Traits of In Vitro Shoot Cultures during Storage

Samples to evaluate the morpho-physiological stage of *S. redliana* ‘Burokvölgy’ in vitro shoot cultures were observed on the first day of storage (three weeks old) and every four weeks during storage. Both the shoot growth (shoot length: SL) and shoot proliferation rate (number of newly developed shoots per explant: SN) were affected by environmental conditions during storage of in vitro shoot cultures.

#### 2.1.1. Shoot Length

**Figure 1:**
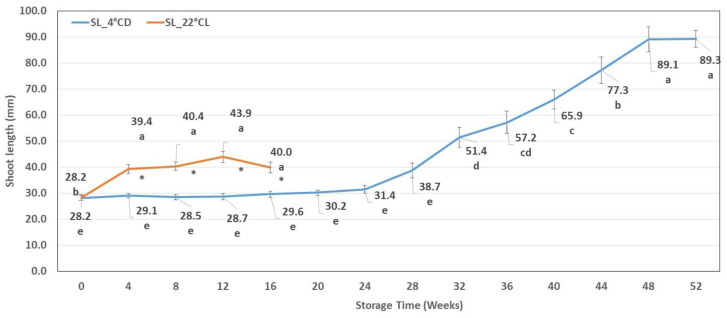
Shoot length (SL) during cold storage (SL_4 °CD) and during storage in the growth chamber (SL_22 °CL). Line graphs present mean values ± SE of SL. Lower case indicates the significant differences between the SL measured after different storage times. * indicates the significant differences between the SL measured after different storage conditions.

No significant shoot growth occurred during the first 24 weeks of the cold storage (at 4 °C and darkness: 4 °CD), and the length of shoots was between 28 ± 5 and 31 ± 7 mm. Shoot elongation started after 24 weeks of cold storage. A period of significant shoot elongation was then observed, which lasted until week 48. The average shoot length reached 89 ± 16 mm. In contrast, intensive shoot growth was detected during the first four weeks of storage at 22 °CL, but no significant changes were observed after that. The average length of the shoots stored at 22 °CL reached just 43 ± 11 mm between weeks 4 and 16, and these shoots were significantly longer than those stored at 4 °CD during the same period. However, the shoots stored at 4 °CD reached a significantly longer shoot length (51 ± 19 mm) from the 32nd week compared to the highest results obtained in the growth chamber (43 ± 11 mm) (Figure 1).

#### 2.1.2. Shoot Number

In the case of the shoot cultures stored at 4 °CD, the degree of shoot proliferation was not proven to be significant during most of the storage period. However, at the 36th and 52nd week, we detected significantly higher shoot numbers compared to the initial state.

**Figure 2:**
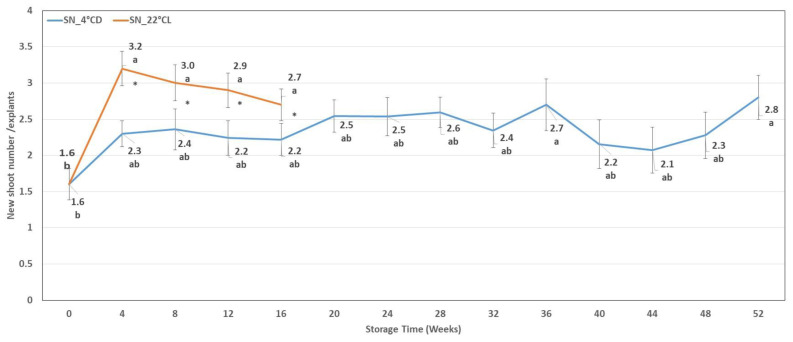
Number of newly developed shoots (SN) during cold storage (SN_4 °CD) and during storage in the growth chamber (SN_22 °CL). Line graphs present mean values ± SE of SN. Lower case indicates the significant differences between the SN measured after different storage times. * indicates the significant differences between the SN measured after different storage conditions.

In shoot cultures stored at 22 °CL, the number of shoots increased significantly in the first four weeks and then did not change significantly in the period between weeks 4 and 16, which was similar to the trend in shoot length. Moreover, in the period between the 4th and 16th week, the degree of shoot proliferation in the cultures stored at 22 °CL was significantly higher compared to those stored at 4 °CD (Figure 2).

### 2.2. Physiological Traits of In Vitro Shoot Cultures during Storage

**Figure 3:**
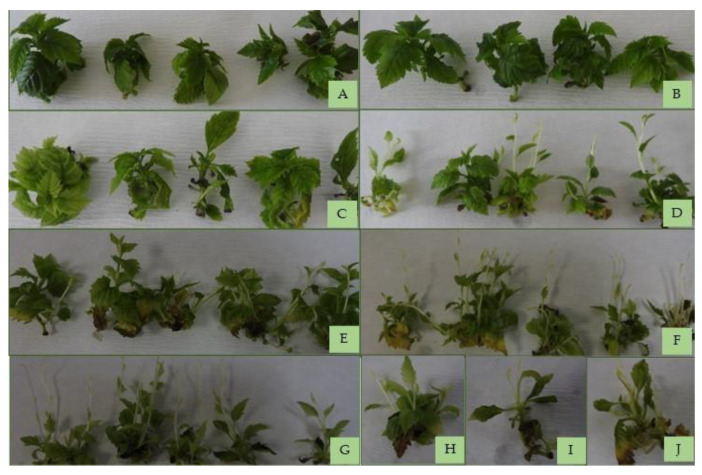
Shoot cultures of *Sorbus redliana* ‘Burokvölgy’ after storage at 4 °CD and darkness for four weeks (**A**), 16 weeks (**B**), 24 weeks (**C**), 36 weeks (**D**), 40 weeks (**E**), 44 weeks (**F**), 48 weeks (**G**), and 52 weeks (**H**–**J**).

**Figure 4:**
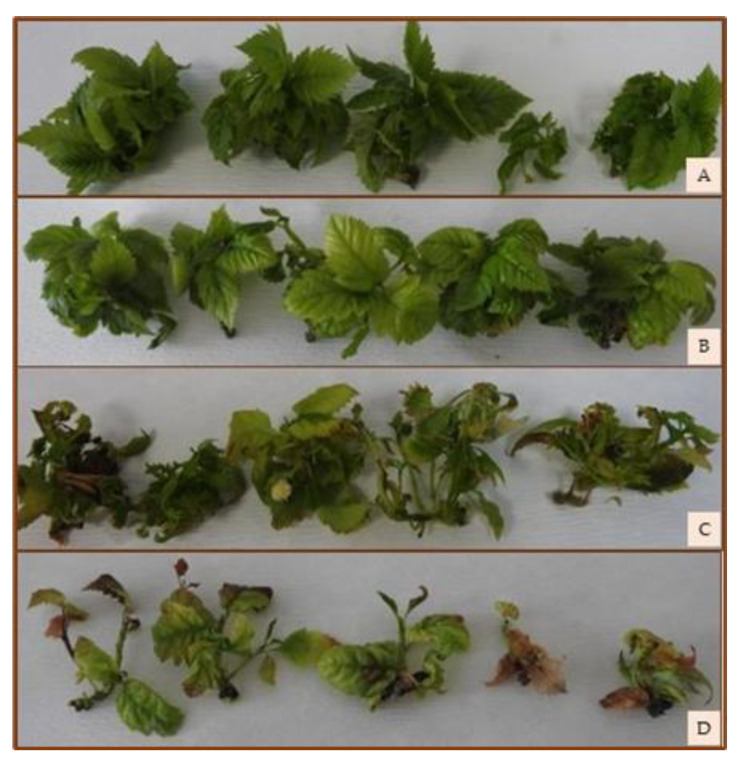
Shoot cultures of *Sorbus redliana* ‘Burokvölgy’ after storage at 22 °CL and 16 h light for four weeks (**A**), 8 weeks (**B**), 12 weeks (**C**), and 16 weeks (**D**).

The rate of hyperhydration was high after 48 weeks of cold storage (up to 60%) and after 12 weeks of storage at 22 °CL (up to 25%). In the case of the shoot cultures stored at 4 °CD, the yellowing of the leaves started after the 24th week, and after the 32nd week, browned tissues also appeared. Around the 28th week, in the phase of intensive shoot growth, etiolated shoots appeared, and after the 36th week, most shoots were already etiolated (Figure 3). After 48 weeks of storage, tissue death appeared on 30% of the shoot cultures. The shoots stored at 22 °CL showed signs of senescence around the 12th week, and extensive browning and tissue death were already common in the 16th week (Figure 4) (Table 1). In general, the etiolation was located on the upper part of shoots, while yellow and brown discoloration tended to occur on the lower part of shoots. All these changes were also reflected in the changes in chlorophyll content.

#### 2.2.1. Chlorophyll Content of Leaves

The chlorophyll content of the leaves was measured every 4 weeks during the experiment. At the start of the experiment, the chlorophyll content of the leafy shoots (three weeks old) was 0.62 and 0.27 mg g^−1^ FW (chl *a* and chl *b*).

Both the chl *a* and chl *b* content decreased significantly during storage regardless of the storage conditions. The chl *a* and chl *b* content of shoots stored at 4 °CD decreased significantly in the first four weeks and then remained roughly at the same level, and no further significant decrease occurred until the end of the 20th week. This period was followed by a phase in which the chl *a* and chl *b* content decreased significantly again. A similar periodic change was observed in the quantitative changes of the total chlorophyll content. In contrast, in the case of shoot cultures stored at 22 °CL, a very significant decrease in both chl *a* and chl *b* content occurred during the first 8 weeks, and the same trend could be seen in the changes in the total chl content.

Comparing the storage conditions, we found that the chl *a* and chl *b* content of the shoot cultures was lower during storage at 22 °CL than during storage at 4 °CD, and in the eighth week, the difference was significant. The shoot cultures stored under the 22 °CL condition had a significantly lower chl *a*/chl *b* ratio than did those cultures stored at 4 °CD (Table 2).

#### 2.2.2. Chlorophyll Fluorescence Results

The average F_v_/F_m_ value measured on the leaves of the starting material (three-week-old shoot cultures) was 0.68. Later, values of F_v_/F_m_ varied between 0.33 and 0.65 as measured on shoots stored at 4 °CD and between 0.61 and 0.71 as measured on cultures stored at 22 °CL.

**Figure 5:**
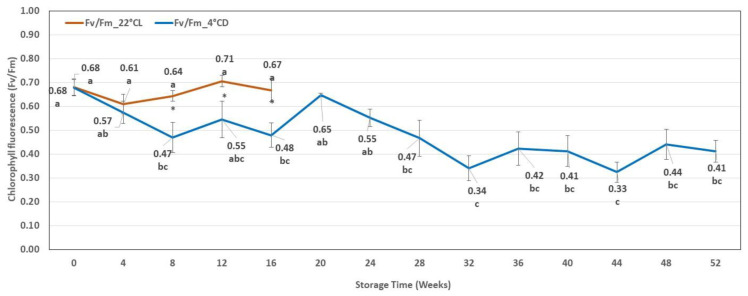
F_v_/F_m_ values measured on shoots after different periods of cold storage (F_v_/F_m_ _4 °CD) and of storage in the growth chamber (F_v_/F_m_ _22 °CL). Line graphs present mean values ± SE of F_v_/F_m._ Lower case indicates the significant differences between the F_v_/F_m_ measured after different storage times. * indicates significant differences between the F_v_/F_m_ values measured after different storage conditions.

In the case of shoot cultures stored at 4 °CD, the F_v_/F_m_ value decreased significantly in the first eight weeks in respect to the initial value, an increased tendency was detected around the 20th week, and then decreased values were measured from the 28th week. In contrast, no significant changes could be detected in F_v_/F_m_ value in shoot cultures kept under the 22 °CL condition. Moreover, in the case of cultures stored at 22 °CL, significantly higher values were measured in the storage period between the 8th and 16th week compared to those stored at 4 °CD (Figure 5).

### 2.3. Number of Usable Explants

**Figure 6:**
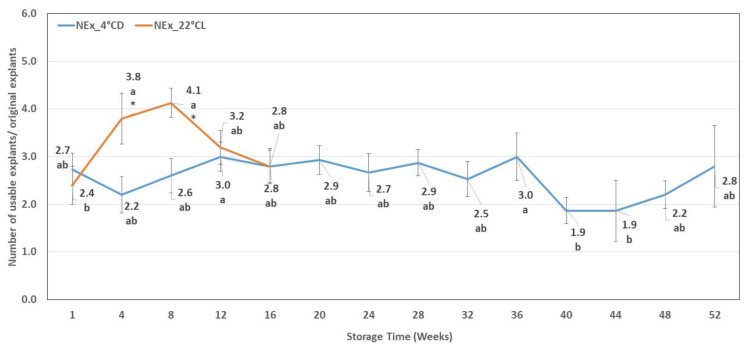
The number of usable explants after different periods of cold storage (NEx_4 °CD) and of storage in the growth chamber (NEx _22 °CL). Line graphs present mean values ± SE of the number of usable explants. Lower case indicates the significant differences between the number of usable explants counted after different storage times. * indicates the significant differences between the number of usable explants after different storage conditions.

Shoot cultures were taken from the starting materials and during storage every four weeks for the purpose of subculturing. The number of new explants obtained per original shoot explant (number of usable explants) was recorded. In the case of shoot cultures stored at 4 °CD, it was almost the same between the 8th and the 32nd week, but it significantly decreased at the 40th week relative to the 36th week. During this period, extremely variable results were observed: there were already long shoots but not in a very good physiological condition, they were etiolated, and a certain amount of tissue death was already noticeable. In the case of shoot cultures kept at 22 °CL, a significant increase was detected in the first four weeks, and then a significant decrease was observed between the 8th and 12th week (Figure 6).

Comparing the two storage conditions, we found that at around the 4th and 8th week, significantly more explants could be obtained from the cultures stored at 22 °CL. In the case of cold storage, the usable explant number per shoot clump varied between 1.9 ± 1.1 and 3.0 ± 1.2, while it varied between 2.4 ± 1.6 and 4.1 ± 1.2 in 22 °CL.

### 2.4. Shoot Proliferation Parameters of Explants from Different Storage Conditions

Explants collected from stored in vitro cultures were cultured under growing conditions (16 h light with 57 µmol m^−2^ s^−1^ PPFD, 22 ± 2 °C) to regenerate new shoots. The length and the number of newly developed shoots was observed after a 4-week period. Both shoot parameters were affected by previous environmental conditions during storage. Lengths of newly developed shoots varied between 23 and 33 mm and between 18 and 23 mm on explants from storage at 4 °CD and 22 °CL, respectively. The number of new shoots varied between 2.7 and 4.7 mm and between 1.9 and 2.8 mm on explants from storage at 4 °CD and 22 °CL, respectively.

#### 2.4.1. Length of Shoots at the End of Subculture Period

**Figure 7:**
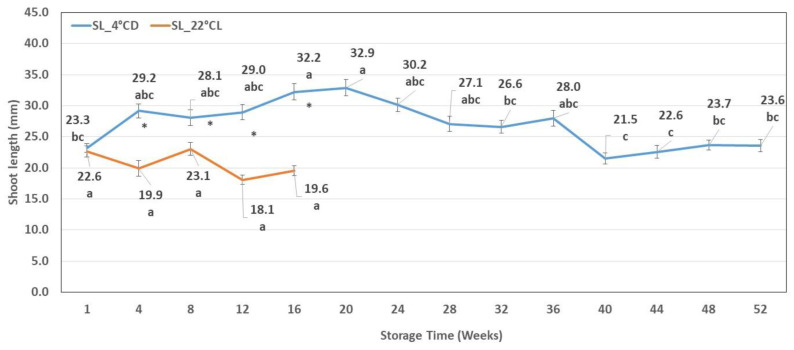
Shoot length (SL) of new shoots developed on subcultured explants from cold storage (SL_4 °CD) and from the growth chamber (SL_22 °CL). Line graphs present mean values ± SE of the SL of new shoots developed from subculture. Lower case indicates the significant differences between the SL measured after different storage times. * indicates the significant differences between the SL measured after different storage conditions.

In the case of explants stored at 4 °CD, we observed that longer shoots (29 mm on average) developed after a storage period of 4–36 weeks compared to a shorter or longer storage (both were 23 mm on average). However, significantly longer shoots developed only when storage duration was 16 to 20 weeks compared to those stored for one week or longer than 40 weeks. No significant differences could be detected after a different period of storage at 22 °CL, whereas the length of new shoots developed on explants from cold storage (4 °CD) was significantly greater than that developed on explants from storage at 22 °CL (Figure 7).

#### 2.4.2. The number of New Shoots at the End of the Subculture Period

In the case of explants from shoot cultures stored at 4 °CD, an increase could be seen after the first four-week storage period, but it was not statistically significant. However, after the 20th week, the proliferative capacity of the explants increased significantly, but later a significant decrease could be observed starting from the 36th week.

**Figure 8:**
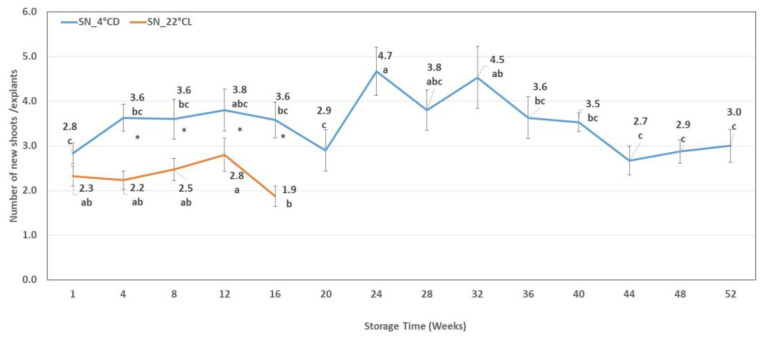
Shoot number (SN) of new shoots developed on subcultured explants from cold storage (SN_4 °CD) and in the growth chamber (SN_22 °CL). Line graphs present mean values ± SE of SN of new shoots developed from subculture. Lower case indicates the significant differences between the SN measured after different storage times. * indicates the significant differences between the SN measured after different storage conditions.

In the case of shoots stored at 22 °CL, a significant increase in the shoot proliferation capacity of explants was not observed until the 12th week. The number of newly developed shoots collected at week 16 was significantly lower than that of explants from 12-week storage. Comparing the shoot proliferation capacity of shoot explants from different storage conditions, we observed that the number of new shoots was significantly lower on explants from 22 °CL than that on those from 4 °CD storage (Figure 8).

**Figure 9:**
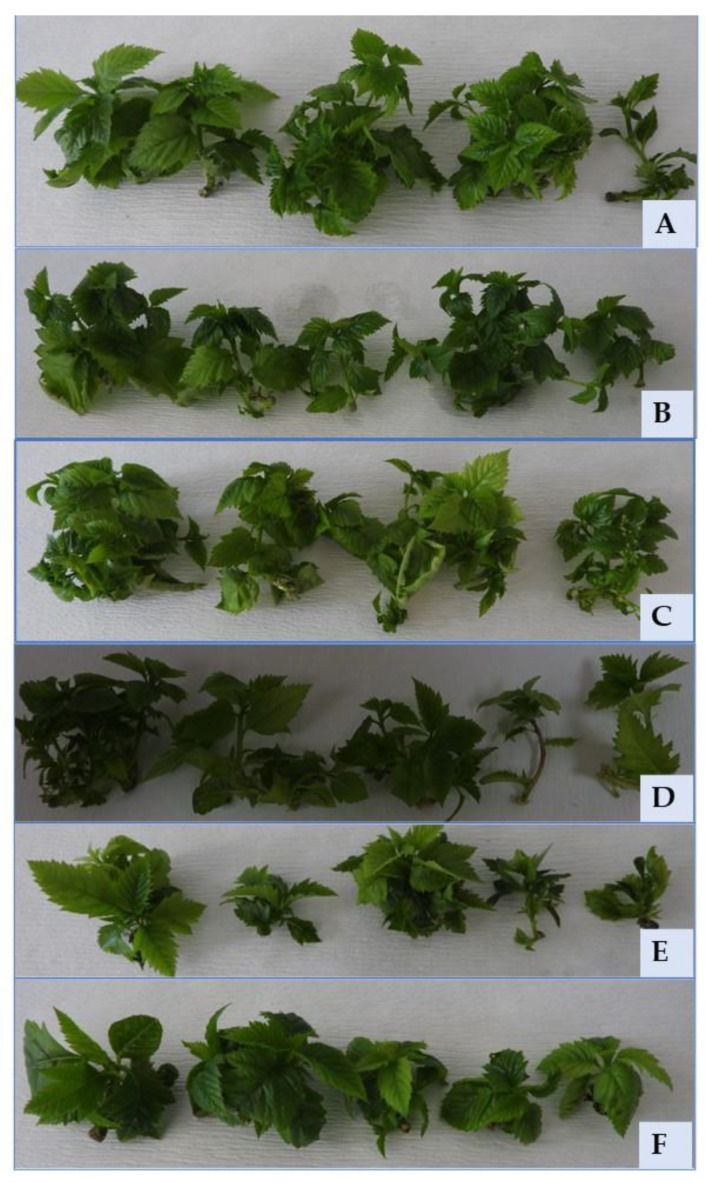
Four-week-old shoot cultures of *Sorbus redliana* ‘Burokvölgy’ developed during the first subculture after storage at 4 °CD for four weeks (**A**), at 4 °CD for 16 weeks (**B**), at 4 °CD for 36 weeks (**C**), at 4 °CD for 52 weeks (**D**), at 22 °CL for four weeks (**E**), and at 22 °CL for 16 weeks (**F**).

However, the physiological state of the subcultured shoot cultures was completely adequate. Although necrotic tissues could be detected on shoots, the slow growth storage condition (4 °C and darkness) prolonged up to 52 weeks did not affect the survival rate (100%) or the success of regrowth during the first subculture of *Sorbus redliana* ‘Burokvölgy’ (Figure 9).

## 3. Discussion

Although maintenance of valuable genotypes in field collections is the most common way to preserve woody plants, the application of in vitro techniques has also been proposed [38]. Responses to cold storage of in vitro cultures of plant species and varieties can be highly varied, and even if they can survive the storage, differences in their regrowth capacity are common.

In our experiments, in vitro shoot cultures of *Sorbus redliana* ‘Burokvölgy’ were stored at 4 °CD for 52 weeks. In general, woody plants of the temperate zone tolerate low storage temperatures well because they can go dormant to survive the winter [39]. Dark conditions can enhance the stability of in vitro culture via the inhibition of development of new tissues, as was observed during a 60-day storage of potato and ginger cultures [18]. Darkness can also enhance the vigor of shoots when stored up to 12 weeks, but in a longer storage period, illumination was found to significantly increase the regrowth ability of chokecherry shoots (*Prunus virginiana*) [40].

Accordingly, we found that all in vitro shoot cultures survived the cold storage at least 52 weeks; however, after an initial dormant period of about 5 months, the shoots began to grow, and the newly developed parts of these shoots were strongly etiolated and elongated.

In another study, newly developed lateral shoots were observed on in vitro shoot cultures of apple (*Malus pumila* Mill., ‘Starkspur Red’) stored at 4 °C after 8 months [41]. In contrast, we did not find an increase in the number of shoots because the growth of lateral buds might have been inhibited further.

As mentioned above, the possibility of slow growth storage in the case of temperate woody plants is based on the ability to adapt to the cold by entering dormancy [28]. This dormant period is usually observed during the first 20 weeks of cold storage, after which intensive shoot growth begins, lasting until about the 40th week, and is followed by a slightly reduced growth period [26]. Thus, growth of woody plants with in vitro cultures during cold storage is a common phenomenon. Similarly, in our experiments, the dormant period of *Sorbus* lasted to 24 weeks, starting approximately between the 4th and 8th week of cold storage, and after this, an intensive shoot growth was observed, which appeared to slow after 48 weeks.

Although we obtained explants from each cold storage period for the first subculture with very high regeneration ability, longer new shoots and higher multiplication rates were detected when shoot cultures were stored from 4 to up to 36 weeks. In a previous study, decreased proliferation was detected in dwarf apple (*Malus domestica*) P2 rootstocks in subculture after 24, 30, and 36 weeks of cold storage (4 °C, dark) [42]. In contrast, in the case of two woody plants (chokecherry and saskatoon berry), the most vigorous regrowth was observed after a 12-month storage period [40].

Moreover, the length of new shoots developed on explants from *Prunus avium* in vitro cultures stored at 2 °C increased as the storage period increased up to nine months [43]. However, the number of newly developed shoots increased only on those explants collected from cold storage lasting shorter than six months, and a longer cold storage resulted in decreased shoot number in the first subculture [43].

In contrast, we did not discover there to be significant differences during the first subculture when explants were obtained from shoot cultures stored for different period durations in the growth chamber (22 °CL). However, the multiplication rates were significantly higher on those explants from cold storage compared to those from the growth chamber when the storage period was longer than four weeks. In contrast, another study reported no significant differences were in the propagation rate of cold-stored *Taraxacum pieninicum* explants compared to that of a nonstored control [44]. Because our control cultures were stored in a growth chamber until they fully aged, the differences may be due to the aged explant sources stored in the growth chamber (22 °CL).

Although all cultures survived the one-year cold storage and proved to be an adequate source of explants for further propagation, signs of damage became visible during the second half of storage. In the shoot cultures stored at 4 °CD, yellow leaves first appeared on the 24th week, and intensive shoot growth began around the 28th week, which resulted in etiolated shoots. By the 32nd week, some brown leaves were also visible, and at the 36th week, all shoots were already etiolated. Necrosis could be observed on approximately 30% of in vitro shoot cultures, especially in the lower part of the shoots after the 48th week. In a study of chokecherry (*Prunus virginiana*), yellow discoloration of lower leaves had no effect on the regrowth capacity [40].

Changes related to aging and damage were also reflected in changes in chlorophyll content. In vivo, during the study of 937 plant species, the total chlorophyll content was found to be very variable, with values ranging from 1.45 to 19.2 mg/g [45]. In our experiments, even the highest total chlorophyll content measured in the absolute control plants (3-week-old *Sorbus* shoot cultures before storage) did not exceed the value of 0.89 mg/g. Both storage conditions resulted in a decrease in chlorophyll content. During storage at 4 °CD, all chl *a* and chl *b* content decreased significantly by the 20th week. In contrast, during storage at 22 °CL, the chl *a* content fell significantly only at the end of the 16th week, whereas the chl *b* chlorophyll content had already decreased significantly at the 8th week. However, from the 8th week, all chlorophyll parameters showed significantly lower values in the 22 °CL storage compared to the 4 °CD storage. The decrease in chlorophyll content during storage at 22 °CL was probably attributable to aging, while the appearance of etiolation during storage at 4 °CD could have caused the decrease in chl content. The chl *a*/*b* ratio did not show significant changes over time in any of the storage conditions. However, significantly lower values were observed in plants stored at 22 °CL starting from the 8th week compared those stored at 4 °CD. Since the light absorption peaks of chl *a* and chl *b* are different (chl *a*: red; chl *b*: blue), the changes in the chl *a*/*b* ratio may indicate the plant’s adaptive response to altered light conditions [45]. In response to a decrease in light intensity, the number of light-harvesting complexes can increase [46]; thus, the chl *b* biosynthesis is expected to become more intense to maximize the light-harvesting capacity due to decreasing illumination [47]. However, in our experiments, low temperature probably inhibited chl biosynthesis as has been observed in many plant species [48].

The correlation between the chlorophyll content of the leaves and the chlorophyll fluorescence parameters is a known phenomenon in the plant kingdom and was also proven in the case of intact *Sorbus aucuparia* plants [49]. Measurement of the photosystem II chlorophyll fluorescence is a very common way to monitor the plant functions since PS II is very sensitive to several stress effects [50]. In general, the F_v_/F_m_ value of the intact C3 type of plant is about 0.83 under optimal conditions [51], and it was found that the F_v_/F_m_ ratio from in vivo plants without any kind of stress varied between 0.7 and 0.8 [52]. Indeed, this value (0.827) was also obtained on intact *Sorbus aucuparia* plants [49]. However, in vitro shoot cultures of *Sorbus redliana* ‘Burokvölgy’ never reached the 0.8 F_v_/F_m_ value, and even the highest value was only 0.706 (measured in control plants stored in growth chamber: 22 °CL), suggesting that in vitro cultivation is an inherently stressful environment [53]. Moreover, we detected a significant decrease in F_v_/F_m_ ratio during the cold storage period, except for around week 20 when we measured an increase. This happened around the time when the rest period ended and shoot growth started. In contrast, research indicates that in vitro shoot cultures stored at 22 °CL do not show any significant changes: the cold stress results in a decreased F_v_/F_m_ ratio under in vivo conditions in woody plants, such as grape [52] and cherry [54], and in herbaceous plants, such as corn [55] and rice [48].

Further studies may help to increase the efficiency and possibly contribute to increasing the storage time. The negative morpho-physiological changes occurring from the second half of the cold storage can perhaps be attributed to the fact that the intensive shoot growth after the end of the rest period resulted in the exhaustion of the cultures. Shoot cultures of *Sorbus*‚ ‘Krasnaja Krupnaja’, grown on medium with high sucrose content (60 g L^−1^) have been successfully stored for up to five years [28]. Further studies including altered medium may help to increase the storage time. Testing other temperatures may also lead to an increase in storage efficiency as in the case of a gooseberry study, in which 2 °C was found to be much more suitable for the storage of shoot cultures than was 4 °C, which was reflected in a lower proportion of necrotic shoots [56].

## 4. Materials and Methods

### 4.1. Establishment and Maintainance of S. redliana In Vitro Cultures and Experimental Conditions

Axillary buds and shoot tips were collected from a dormant mother tree in the arboretum of the Hungarian University of Agriculture and Life Sciences in 2015. Surface sterilization started with soaking in tap water with Tween 80 for 2 h, followed by HgCl_2_ (0.5%) and ethanol (70%) treatment for five minutes, and finished by a rinse in sterile distilled water (three times). The medium for initiation consisted of Benczúr-Márta (BM) macro elements [57], Heller microelements [58] supplemented with Murashige-Skoog (MS) [31] vitamins, and 1.4 µM of 6-benzyladenine riboside (BAR) [59].

The maintenance and propagation of in vitro shoot cultures of *S. redliana* ‘Burokvölgy’ for the experiments were performed using MS medium, which was supplemented with 2.8 µM of BAR, 0.6 µM of gibberellic acid (GA_3_), 1.48 µM of indole-3-butyric acid (IBA), 100 mg L^−1^ of myo-inositol, 3% sucrose, and 0.7% agar-agar. Before autoclaving, the pH was adjusted to 5.8. Five shoot explants (stem segments about 15 mm with at least one node) per 400 mL Kilner jar were cultured in a growth chamber (16/8 h light/dark with 57 µmol m^−2^ s^−1^ PPFD, 22 ± 2 °C) for 3 weeks. Then, 100 jars were transferred into another room (4 °CD) for cold storage periods of 4–52 weeks, while the remaining 56 jars were left in the growth chamber as controls (22 °CL) for a further 16 weeks (total 19 weeks) until they were completely senesced. Samples were taken every four weeks to monitor changes induced by different storage conditions.

### 4.2. Morphological Observations on Stored Shoot Cultures

The number of new shoots per explant were counted, and their length (mm) was measured and recorded at the beginning of storage when the cultures were 3 weeks old and every four weeks thereafter.

### 4.3. Measurements to Detect Physiological Changes in Stored Cultures

In addition to the above-mentioned measurements, visual observation was conducted after every period of storage (every four weeks), and the explants were transferred to fresh medium. The main qualitative parameters of stored shoots were studied, including hyperhydration, intensity of green color, the presence of discoloration or tissue necrosis, and etiolation. Recording of changes consisted of the presence or lack of these symptoms independent of their extension, and then the percent (%) of the newly developed shoots was calculated. 

#### 4.3.1. Measurement of the Chlorophyll Content in Leaves

A spectrophotometric method [60] was used to determine the quantity of chlorophyll a and b (chl *a* and chl *b*) of the leaves. The absorbance of solutions was measured at 653, 666, and 750 nm with a 6705 UV/Vis spectrophotometer (Jenway 6705, VWR International Kft, Debrecen, Hungary). Calculated chl content (mg g^−1^ fresh weight) was obtained after determination of chl *a* and chl *b* values as follows (after excluding the effect of solution turbidity measured at 750 nm) [60]:

chl *a* = 17.12 A666 − 8.68 A653

chl *b* = 32.23 A653 − 14.55 A666

chl *a* + chl *b* = 2.57 A666 + 23.6 A653

#### 4.3.2. Measurement of Chlorophyll Fluorescence

Chl fluorescence was measured with an OS5p modulated fluorometer (Opti-Sciences, Hudson, NH, USA) on fully developed leaves. One leaf per plant was selected, and this was repeated for ten independent plants per treatment (on 2 plantlets per jar, from 5 jars). After a 30-min-long dark adaptation of leaves (by using leaf clips), the F_v_/F_m_ protocol was used to measure maximum quantum yield (estimate of the maximum ratio of absorbed quanta used in the PSII reaction center) [61]. Maximum (F_m_) and minimum (F_o_) were measured in dark-adapted leaves after 0.8 s for the saturation pulse (35 W halogen lamp with 690 nm short pass filter according to the OS5p User’s Guide). The potential quantum efficiency of PSII (F_v_/F_m_ ratio), which is an indicator of photosynthetic performance, was calculated by the built-in software (Opti-Sciences Inc. 603-883-4400) of the fluorometer [62].

#### 4.3.3. Number of Usable Explants after Storage

Usable shoot explants from another 3 jars were counted to help calculate the required number of shoot cultures for storage in the future. These explants (stem segments about 15 mm with at least one node) were subcultured. Moreover, only those shoots which did not show yellowing, browning, or necrosis on their upper part were subcultured. In the case of etiolated shoots, the weak, thinned upper part of shoots was not used for subculture. Before statistical analysis, the number of usable explants was calculated as follows:

NEx = the number of usable explants per jar/the number of initial explants per jar

### 4.4. Morphological Observations on Subcultured Shoot Cultures

Shoot explants were subcultured onto fresh medium and transferred to the growth chamber for a 28-day period to test the ability of regrowth (the medium and growing conditions were the same as those used for the shoot multiplication). The observations were the same as those described in Section 4.2.

### 4.5. Data Collection and Analysis

The shoots included in morphological observations (shoot length, number of new shoots) were developed on a total of 25 explants from 5 jars. Samples were collected as five replications from five different jars (2 subsamples per jar) for measurements (chlorophyll content, chlorophyll fluorescence). Statistical analysis of the data was carried out using one-way analysis of variance (ANOVA) and followed by Tukey’s test to reveal significant differences between the means at a level of *p* ≤ 0.05 with SPSS software version 21.0 (SPSS^®^) for Windows.

## 5. Conclusions

This is the first report on slow growth storage of *S. redliana.* In the current study, it was found that in vitro shoot cultures of *S. redliana* can be stored safely at 4 °C in the dark for 52 weeks on MS medium supplemented with 3% sucrose and 2.8 µM of BAR, 0.6 µM of GA_3_, and 1.48 µM of IBA. During this period, the survival of stored shoots was 100%, and thus stored explants can be regrown at any time with an efficient multiplication rate. However, considering the performance of subcultured explants, we advise waiting for the end of the dormant period. Moreover, after week 32, the number of usable explants becomes unreliable, and after the 48th week, some damage may already be detectable on the stored plants, with the performance of the first subcultured explants being decreased. However, in vitro shoot cultures were completely senescent at 19 weeks (they were three weeks old at the beginning of the 16-week storage period) under normal conditions (22 °CL). As the most frequently used subculture period four weeks, it is possible to avoid at least three subcultures with significant savings in raw materials, costs, and working time.

## Figures and Tables

**Table 1:** The rate of physiological changes observed for the in vitro shoot culture of *S. redliana* ‘Burokvölgy’ stored under cold condition (4 °CD) and the growth chamber (22 °CL).

Storage Weeks	Hyperhydration (%)	Discoloured Leaves (%)	Necrotic Tissues (%)	Etiolated Shoots (%)
		4 °CD		
0	6.2	0.0	0.0	0.0
4	15.9	0.0	0.0	0.0
8	11.2	0.0	0.0	0.0
12	24.5	0.0	0.0	0.0
16	16.5	0.0	0.0	0.0
20	21.4	0.0	0.0	0.0
24	25.6	0.0	0.0	0.0
28	34.2	4.6	4.6	29.1
32	37.8	45.9	0.0	51.6
36	38.5	45.8	0.0	71.5
40	29.2	58.2	0.0	90.0
44	51.1	64.5	5.2	100.0
48	60.7	73.8	28.7	100.0
52	46.6	87.1	31.4	87.4
		22 °CL		
0	3.5	0.0	0.0	0.0
4	12.7	0.0	0.0	0.0
8	14.0	14.4	0.0	0.0
12	25.0	79.3	16.5	0.0
16	27.2	100.0	85.0	0.0

**Table 2:** Chlorophyll content (Chl *a*, Chl *b*, and Chl*a*/*b*) in the in vitro leaves of *S. redliana* ‘Burokvölgy’ under storage conditions of 4 °CD and 22 °CL. Values are expressed as the mean ± SE of chlorophyll content. Lower case indicates the significant differences between the chlorophyll content measured after different storage times. * indicates the significant differences between the chlorophyll content of leaves from different storage conditions.

Weeks in Storage	Chl *a*		Chl *b*		Chl *a/b*	
	mg g^−1^ Fresh Weight					
	4 °CD	22 °CL	4 °CD	22 °CL	4 °CD	22 °CL
0	0.62 ± 0.06 a	0.62 ± 0.06 a	0.27 ± 0.02 a	0.27 ± 0.03 a	2.29 ± 0.03 ab	2.29 ± 0.03 a
4	0.56 ± 0.03 b	0.47 ± 0.03 b	0.24 ± 0.01 b	0.22 ± 0.01 b	2.38 ± 0.03 ab	2.16 ± 0.02 a
8	0.49 ± 0.02 bc *	0.28 ± 0.02 bc	0.20 ± 0.01 b *	0.13 ± 0.01 c	2.45 ± 0.03 ab *	2.09 ± 0.03 a
12	0.54 ± 0.02 b *	0.31 ± 0.03 bc	0.21 ± 0.01 b *	0.14 ± 0.01 c	2.54 ± 0.02 ab *	2.29 ± 0.04 a
16	0.53 ± 0.02 b *	0.22 ± 0.02 c	0.21 ± 0.01 b *	0.10 ± 0.01 c	2.50 ± 0.03 a *	2.30 ± 0.03 a
20	0.46 ± 0.02 bc	-	0.17 ± 0.01 bc	-	2.63 ± 0.02 ab	-
24	0.33 ± 0.02 cd	-	0.13 ± 0.01 cd	-	2.59 ± 0.04 ab	-
28	0.21 ± 0.02 de	-	0.08 ± 0.01 de	-	2.48 ± 0.04 ab	-
32	0.27 ± 0.04 de	-	0.11 ± 0.01 de	-	2.54 ± 0.04 ab	-
36	0.21 ± 0.02 de	-	0.08 ± 0.01 de	-	2.57 ± 0.04 ab	-
40	0.18 ± 0.02 de	-	0.07 ± 0.01 de	-	2.56 ± 0.04 ab	-
44	0.09 ± 0.01 e	-	0.04 ± 0.004 e	-	2.27 ± 0.03 b	-
48	0.15 ± 0.02 e	-	0.06 ± 0.01 de	-	2.47 ± 0.05 ab	-
52	0.14 ± 0.02 e	-	0.06 ± 0.01 de	-	2.50 ± 0.05 ab	-

## Data Availability

Data are contained within the article.
